# Self-reported handwashing frequency among pet and non-pet owners in the German adult population in 2023: a post-pandemic replication study

**DOI:** 10.1186/s12889-026-27235-1

**Published:** 2026-04-07

**Authors:** Karolin M. E. Nettelrodt, Thomas von Lengerke

**Affiliations:** Department of Medical Psychology (OE 5430), Hannover Medical School (MHH), Carl-Neuberg-Str. 1, Hannover, 30625 Germany

**Keywords:** Hand hygiene, Overestimation bias, COVID-19 pandemic impact, Compliance, Infection prevention/control, Population surveillance

## Abstract

**Background:**

In a previous analysis of data from four surveys of the German adult population conducted in the 2010s, i.e., 2012, 2014, 2017 and 2019, substantial differences between pet and non-pet owners had been found in terms of higher self-reported handwashing compliance among non-pet owners. This difference was especially pronounced for the indication “after touching animals”. Meanwhile, the COVID-19 pandemic has brought increased attention to infection prevention and control regarding interactions of humans with animals, including pets. Thus, it seems reasonable to assume that compliance rates may have changed to a certain degree. Using data from a survey conducted in post-pandemic 2023, our aim was to test this assumption by replicating the previous analyses and comparing pre- and post-pandemic compliance and reasons for non-compliance.

**Methods:**

Data from a cross-sectional computer-assisted telephone survey of the German population from 16 to 85 years, conducted in the summer of 2023, was analyzed (*N* = 3,597). For nine different indications, handwashing compliance was defined as stating to (almost) always wash one’s hands in a given situation. Participants stating to rarely or (almost) never wash their hands were asked for possible reasons. The pre-pandemic data to which the present data is compared comes from the previous surveys conducted in 2012, 2014, 2017 and 2019, which had used the same methodologies. In statistical analyses, Chi²-tests, the Cohen’s d indicator, and multiple logistic regressions were used.

**Results:**

Across these time periods, pet owners were less likely to report handwashing after touching animals (35.5% pre-pandemic vs. 30.0% post-pandemic) in contrast to non-pet owners (55.7% pre-pandemic vs. 55.9% post-pandemic). The effect was found to be independent from potential confounders (*p* < 0.001). In contrast, compliance rates of pet and non-pet owners increased for other indications, in particular those associated with infection prevention measures highlighted in the context of the pandemic, e.g., before visiting someone weakened by disease. “Feeling that it is not necessary” remained the most confirmed reason for not washing hands among pet and non-pet owners (78.7% vs. 66.5%, d = 0.34).

**Conclusion:**

Taken together, results suggest that pet owners seem to be more compliant in situations where handwashing serves the protection of other people. Thus, as one facet of promoting pandemic preparedness, more risk communication may be needed on how and why washing one’s hands after touching animals, including pets, is about protecting other people and oneself from infections.

**Supplementary Information:**

The online version contains supplementary material available at 10.1186/s12889-026-27235-1.

## Highlights

• Little is known about how the COVID-19 pandemic has affected pet owners’ infection preventive behaviors when caring for their pets, e.g., handwashing in different situations.

• Before the pandemic, pet owners reported significantly lower handwashing compliance after touching animals than non-pet owners.

• After the pandemic, pet and non-pet owners differed more widely than before due to decreased compliance among pet owners.

• The results clearly indicate that the pandemic has affected pet and non-pet owners’ handwashing compliance differentially.

• Pet owners may benefit from intensified educational interventions on handwashing as a behavior which serves purposes of self-protection.

**Supplementary Information:**

The online version contains supplementary material available at 10.1186/s12889-026-27235-1.

## Introduction

Pet ownership can have beneficial health-related effects, e.g., increased physical activity, reduced loneliness, anxiety and depression, enhanced emotional well-being, improved quality of life [1], and reduced risks of cardiovascular disease [2]. At the same time, close contact with pets is associated with health risks, particularly with regard to the transmission of pathogens [3]. Overall, about 60–70% of infectious diseases in humans are of zoonotic origin [4, 5]. Compared to other animal hosts, e.g., farm animals, pets have received little attention regarding associated zoonotic risks and their prevention [3, 6]. Yet, evidence suggests that substantial proportions of zoonotic transmission and diseases are associated with pets, e.g., salmonellosis with dogs and reptiles [3, 6–16]. In this context, evidence-based hygienic measures such as handwashing are promoted by public health recommendations (in Germany, e.g., by the Federal Institute of Public Health [17])

During the coronavirus disease (COVID-19) pandemic it has become evident that COVID-19 is better classified as an infectious disease of animal origin with human-to-human transmission rather than an anthropozoonosis, i.e., a zoonosis with animal-to-human transmission [18–21]. Nevertheless, the pandemic has undoubtedly put a spotlight on infection prevention and control measures, inter alia those related to human-animal relationships, including pets. In April 2022, 56% of the adult population in England agreed that washing one’s hands after touching pets is important in order to prevent the spread of COVID-19 [22]. This was at odds with both UK government advice at the time and scientific evidence. In contrast, earlier in the pandemic, in autumn 2020, 80% of US pet owners were not concerned to contract SARS-CoV-2 from their pets [23]. A tracking survey by the UK Food Standards Agency with eight waves from 2020 to 2022 showed a rather stable development in self-reported handwashing of pet owners after contact with animals including pets (36% in July 2020 versus 32% in January 2022, with a peak at 40% in October 2020). No data on non-pet owners’ handwashing compliance after contact with animals was reported. However, population-wide compliance with handwashing in other situations decreased as the pandemic progressed: e.g., for “when returning home” rates were 61% in July 2020 and 47% in January 2022 [24]. Thus, it would be instructive to compare the developments in pet owners’ handwashing compliance after animal contact with that of non-pet owners. Yet, we are not aware of studies reporting any relevant comparisons, in particular for the post-pandemic time period, i.e., after May 5, 2023.

Against this background, we follow up on a pre-COVID-19 analysis of differences between pet and non-pet owners in Germany regarding handwashing behavior [25]. We had used data from four surveys (2012-19) which had been commissioned by the German Federal Centre for Health Education (Bundeszentrale für gesundheitliche Aufklärung, BZgA; now German Federal Institute of Public Health [Bundesinstitut für Öffentliche Gesundheit, BIÖG]). Overall, among nine handwashing indications, the largest difference by far was found for “after touching animals”: 35.5% of pet owners stated to (almost) always wash their hands in this situation, versus 55.7% of non-pet owners. This difference was independent of gender, age, educational background, migration background, children under 16 years in the household, chronic disease, and working in healthcare. Additionally, among those reporting to (almost) never or rarely washing their hands after touching animals, 79.0% of pet owners felt that it is not necessary to wash their hands in such situations (non-pet owners: 67.1%). This was the only reason for non-adherence which pet owners agreed to more frequently than non-pet owners. Again, the difference was independent of potential confounders.

There are reasons to assume that handwashing behavior of pet owners may have increased or at least remained stable during the pandemic: there is evidence that pets may acquire COVID-19 from direct contact with infected owners [19, 26], and that pet owners were concerned for their pets because of this transmission route [27, 28]. Presumably, non-pet owners’ compliance rates rather decreased (as in the mentioned data from UK [24]). Thus, the difference in handwashing compliance after touching animals might diminish.

In the summer of 2023, a general population survey analogous to those between 2012 and 2019 was conducted, again commissioned by the BZgA [29]. In the respective report published on the BIÖG website, it was only stated that respondents without pets washed their hands (almost) always after contact with animals more often than pet owners (55% vs. 29%) [29]. This gave us the opportunity to replicate the pre-pandemic analyses [25] with data gathered right after the pandemic across all nine indications, and thus more comprehensively compare pre- and post-pandemic handwashing of pet and non-pet owners.

## Methods

The methods used correspond to those in [25], with the most important information presented in the following for reasons of clarity.

### Study design, setting, and participants

Like the 2012-19 surveys [30–33], the survey commissioned by the BZgA in 2023 was administered by the Forsa Institute for Social Research and Statistical Analysis as a cross-sectional, representative, computer-assisted telephone interview survey of the general population in Germany aged 16–85 years. The 2023 sample was increased to include sufficient sub-samples of parents with children under the age of 16 in the household. Thus, it included *N* = 4,001 interviews [29]. The data was provided to us by the BIÖG upon our request (Andrea Rückle, personal communication, May 6, 2025). We used the same exclusion criteria as in [25]. Therefore, *N* = 372 interviews did not enter statistical analyses: one respondent who stated not washing their hands even once a day, 32 respondents who did not provide any information about handwashing frequency or duration, 311 respondents who did not provide information on handwashing frequency in the nine indications to be examined in our analysis, 24 respondents without information on their age, four respondents without information on their gender, and zero respondents due to missing information on pet ownership. Eventually, *N* = 3,629 respondents were included. In statistical analyses, data was weighted to compensate for sampling biases inherent in the differential selection probabilities for the two sampling frames (fixed-line and mobile) and the oversampling of parents with children under the age of 16. Weighting resulted in a sample with rounded total of *N* = 3,597.

For comparison, we include below the results based on the pooled data set of the four surveys conducted in 2012, 2014, 2017 and 2019 [25, 30–33]. The set consisted of *N* = 16,993 interviews, of which *N* = 15,682 interviews were included in the analysis (weighted *N* = 15,559). A detailed description of the study design, which applied to the 2023 survey as well, can be found in [25].

### Measures: survey items and compliance indices

To replicate the analysis in [25], we operationalized and coded all variables as we did with the pooled data from the 2012 to 2019 surveys. In the following sections, our translations of the original German survey items are provided. The original items are available from the corresponding author and in [29–33]. For all items, the answer categories “I don’t know” and “Not specified” were not presented in the interview but coded either if the respondent gave a respective answer by him- or herself, or responded in a way that could, after clarification, be fitted validly into one of these categories by the interviewer.

#### Handwashing compliance and reasons for non-compliance

Operationalization of the frequency of handwashing given nine indications used the same item as described in [25]: “How often do you wash your hands in each of the following situations, i.e., “never/almost never”, “seldom”, “mostly”, or “always/almost always”?” Indications were “Before eating”, “After touching animals”, “After handshaking”, “Before handling food”, “After coming home from outside”, “After using the toilet”, “After blowing one’s nose or coughing in one’s hand”, “After being with someone who had the flu, a gastrointestinal disease, or a similarly contagious disease”, and “Before visiting someone who is weakened by disease”. Following [25, 34], handwashing compliance was defined for each variable as “almost always or always”.

As in the 2017 and 2019 surveys, participants in the 2023 survey who reported to “never or almost never” or “rarely” wash their hands given an indication were presented a list of five possible reasons for this behavior and asked if each reason applied to them or not. The proposed reasons were “I feel that it is not necessary”, “I do not have an appropriate washing facility available”, “I do not think of it, or forget it”, “It takes too long” and “Others might consider it inappropriate”. Multiple selections were possible.

#### Socio-demographics

Indexing algorithms followed socio-demographic standards by the German Federal Statistical Office [35] and are available upon reasonable request from the corresponding author and in [25, 34]. The items for gender, age, owning a pet, having a chronic disease, having children under 16 years read the same as in the previous surveys, as well as the items we used to code educational and migration background (for details see [25]). In the 2012 to 2019 surveys, those respondents stating they were employed, in vocational training, higher education or on parental/maternal leave were asked if they personally worked in the medical field with patient contact. Answering “yes” was coded as “currently working in healthcare”. In the 2023 survey, these respondents were asked if they belonged to one of the following occupational categories: “medical field or physicians, respectively”, “(kindergarten) teacher or other educational profession”, “police or fire department”, “public transport”, “retail sales for basic needs”. Answering “yes” to the first option was coded as “currently working in healthcare”.

### Statistical analysis

As in [25], Chi-Square-tests with odds ratios (OR) and 95%-confidence intervals (95%-CI) were used to test for differences between pet and non-pet owners. Breslow-Day-tests were used to test for heterogeneity of the ORs across surveys (2023 vs. 2012-19). P-values < 0.05 were considered to signify statistical significance. Additionally, ORs were transformed into Cohen’s d-coefficients [36] in order to assess effect sizes of the differences.

Multiple logistic regression analyses were conducted to adjust for socio-demographic and other potential confounders of the associations of pet ownership with self-reported handwashing and with the reasons for (almost) never or rarely washing one’s hands. These variables were those being associated with pet ownership, i.e., gender, age, educational background, migration background, having children < 16 in household, having a chronic disease, and currently working in healthcare. ORs from multiple regressions were transformed into Cohen’s d-coefficients [36] as effect sizes. IBM SPSS Statistics v29 was used.

## Results

### Sample description

Table 1 shows that in the 2023 survey, *N* = 1,358 (37.8%) reported to own a pet. Compared to non-pet owners, they are significantly more likely to be of age 59 or younger (80.2% vs. 63.8%), to have children under 16 in their household (31.3% vs. 19.0%) and to currently work in healthcare (10.9% vs. 7.3%). However, pet owners are significantly less likely to have a higher educational background (35.2% vs. 41.2%) and a migration background (19.8% vs. 22.9%).

**Table 1:** Characteristics of the 2023 survey sample, overall and stratified for non-pet owners and pet owners

	Total *N* = 3,597 (100%)		Non-pet owners (*N* = 2,239, 62.2%)		Pet owners (*N* = 1,358, 37.8%)		Chi²-test****	
	N*	%**	*N*	%***	*N*	%***	Chi²	*p*
*Gender*							0.2	0.636
Men	1,777	49.4%	1,113	49.7%	664	48.9%		
Women	1,820	50.6%	1,126	50.3%	694	51.1%		
*Age (in years)*							111.5	< 0.001
16–29	697	19.4%	411	18.4%	286	21.1%		
30–44	869	24.2%	501	22.4%	368	27.1%		
45–59	950	26.4%	516	23.0%	434	32.0%		
60–85	1,081	30.1%	811	36.2%	270	19.9%		
*Educational background* ^a^							42.5	< 0.001
Higher	1,353	39.0%	895	41.2%	458	35.2%		
Medium	1,097	31.6%	600	27.6%	497	38.2%		
Lower	1,023	29.5%	678	31.2%	345	26.5%		
*Migration background*							4.7	0.031
No	2,816	78.3%	1,727	77.1%	1,089	80.2%		
Yes	781	21.7%	512	22.9%	269	19.8%		
*Children under 16 in household*							70.5	< 0.001
No	2,733	76.4%	1,803	81.0%	930	68.7%		
Yes	845	23.6%	422	19.0%	423	31.3%		
*Children age 5 or younger in household*							0.3	0.597
No	3,221	90.3%	2,011	90.5%	1,210	90.0%		
Yes	346	9.7%	211	9.5%	135	10.0%		
*Chronic disease* ^*b*^							3.0	0.085
No	2,459	68.4%	1,507	67.4%	952	70.2%		
Yes	1,134	31.6%	729	32.6%	405	29.8%		
*Currently working in healthcare* ^*b*^							14.1	< 0.001
No	3,282	91.3%	2,074	92.7%	1,208	89.1%		
Yes	311	8.7%	163	7.3%	148	10.9%		

* Any data not adding to the total is due to missing values ** Column percentages *** Row percentages **** Pet ownership by row variable

^a^ Higher educational background equals upper secondary school, middle educational background equals intermediate school, and lower educational

background equals secondary general school

^b^ Pertains to the survey participants personally

Table A.1 in the appendix provides a demographic comparison of the pre-pandemic samples (2012-19) and the post-pandemic sample (2023). The 2023 sample was significantly more likely to be of age 60 or older (30.1% vs. 28.0%), to have a higher educational background (39.0% vs. 32.5%), to have a migration background (21.7% vs. 20.1%), to have a chronic disease (31.6% vs. 28.9%), but significantly less likely to be currently working in healthcare (8.7% vs. 9.5%). No significant difference was found for pet ownership (37.8% vs. 36.9%, *p* = 0.335).

### Pet owners’ and non-pet owners’ self-reported handwashing behavior in different situations

**Fig. 1:**
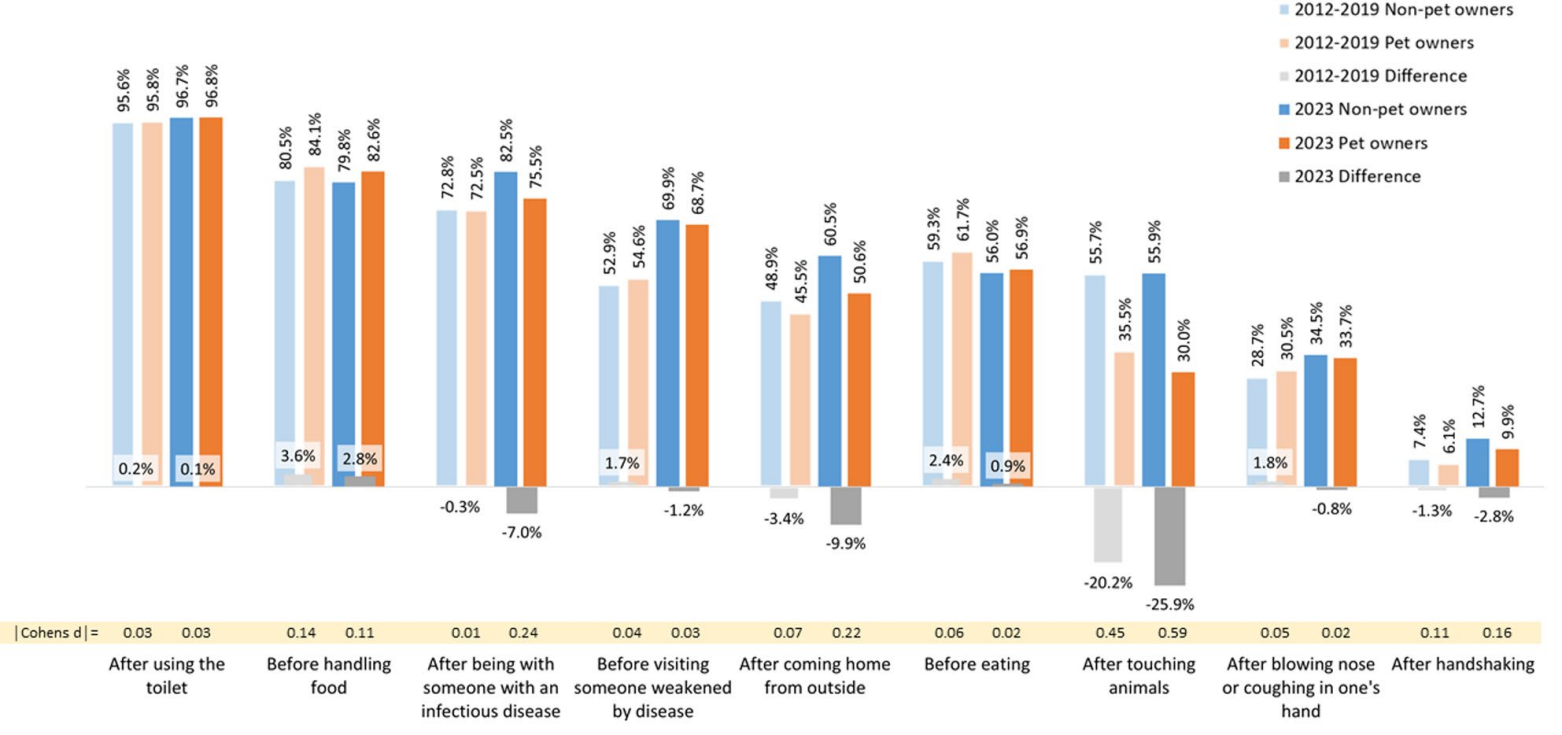
Self-reported handwashing compliance of pet owners and non-pet owners as proportions of those reporting to always or almost always wash their hands in nine different indications (in %). Note: Data from 2012-19 originally published in [25]

Figure 1 shows the compliance rates of pet and non-pet owners for the nine indications for handwashing. They are arranged from left to right in descending order according to the total compliance rates of the 2023 sample (see Table A.2 in the appendix). Data from the previous analysis [25] is presented in lighter colors, and the differences between pet and non-pet owners are presented in grey. In comparing the pre- and post-pandemic compliance rates of pet and non-pet owners, some increases are noticeable. In 2023, 69.9% of non-pet owners and 68.7% of pet owners reported to (almost) always wash their hands before visiting someone weakened by illness, compared to 52.9% and 54.6% in 2012-19. Similarly, compliance rates for “after coming home from outside”, “after being with someone with an infectious disease”, “after blowing nose or coughing in one’s hand” and “after handshaking” increased among pet and non-pet owners.

In both pre- and post-pandemic data, differences between pet and non-pet owners’ compliance rates range in one-digit numbers, except for the indication “after touching animals”. Here, the difference was − 20.2% pre- and − 25.9% post-pandemic. This was mainly due to a decrease in pet owners’ compliance rate (35.5% vs. 30.0%), whereas non-pet owners’ compliance rate remained stable (55.7% vs. 55.9%). Differences for “after being with someone with an infectious disease” and “after coming home from outside” also increased in favor of non-pet owners, due to larger increases in their compliance rates compared to pet owners (see Fig. 1). Breslow-Day-tests revealed that the difference in these three situations differed significantly between pre- and post-pandemic data (see Table A.2). By conventional classifications of effect sizes in terms of Cohen’s d, which were calculated based on the odds ratios, all differences are trivial (d < 0.20) with three exceptions: A small effect was found for “after coming home from outside” and “after being with someone with an infectious disease” (d = 0.22 and d = 0.24, respectively). The effect for “after touching animals” is medium (d = 0.59) and exceeds the pre-pandemic value (d = 0.45).

Multiple logistic regressions show that the effect of pet ownership on handwashing compliance “after touching animals” is independent of gender, age, educational background, migration background, having children under the age of 16 in the household, having a chronic disease and working in healthcare (see appendix, Table A.3). The adjusted odds ratio for pet ownership is OR = 0.34 (95%-CI: 0.29; 0.40, *p* < 0.001, d = 0.59) which is similar to the pre-pandemic results (OR = 0.43, 95%-CI: 0.40; 0.46, *p* < 0.001, d = 0.47) [25].

### Pet and non-pet owners’ reasons for rarely or (almost) never washing their hands after touching animals

Of the pet owners 45.1% reported to rarely or (almost) never wash their hand after touching animals, compared to 17.8% of the non-pet owners. Regarding the list of five possible reasons presented to these *N* = 1,010 respondents, total rates ranged from 73.9% for “I feel that it is not necessary” to 8.6% for “Others might consider it inappropriate”. These rates differ only slightly from the pre-pandemic rates, with the largest difference of − 5% for “Others might consider it inappropriate” (2012-19: 13.6%; see Table A.4 in the appendix).

**Fig. 2:**
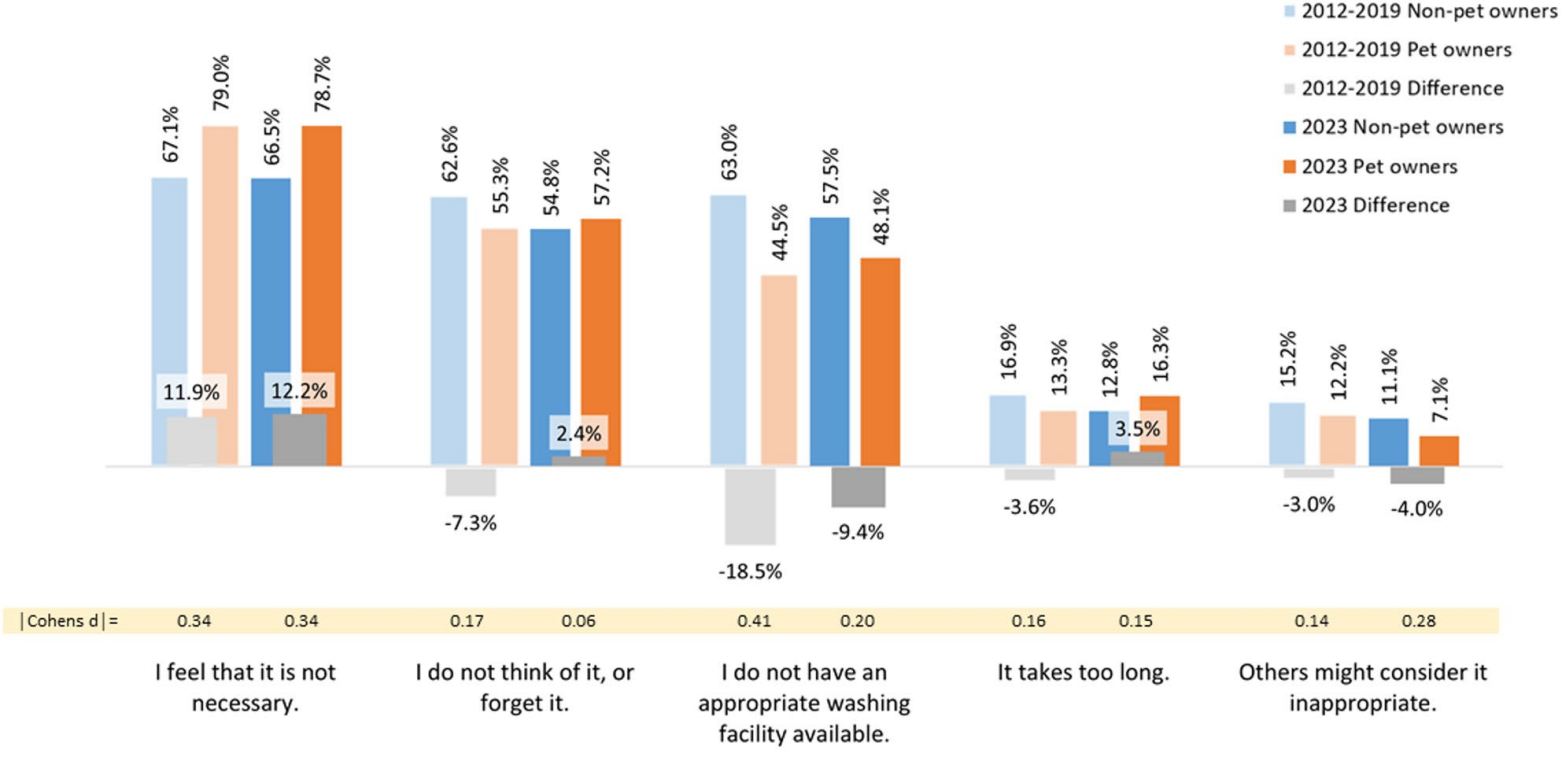
Reasons for self-reportedly never, almost never or rarely washing one’s hands among pet owners and non-pet owners (as proportions – in % – of those confirming each reason). Note: Data from 2012 to 19 originally published in [25]

As Fig. 2 shows, pet owners were 12.2% more likely than non-pet owners to explain their non-compliance with “feeling that it is not necessary” (2012-19: + 11.9%). While pre-pandemically, pet owners were less likely to conform to the other four reasons proposed, in 2023 pet owners were more likely to confirm “I do not think of it, or forget it” and “It takes too long” (+ 2.4% and + 3.5%, respectively). Post-pandemically, both groups differed less widely in terms of agreeing with “I do not have an appropriate washing facility available” than pre-pandemically (2023: − 9.4%, 2012-19: − 18.5%). Accordingly, Breslow-Day-tests show significant heterogeneity of the odds ratios from pre- and post-pandemic data for “I do not think of it, or forget it” (*p* = 0.012), “I do not have an appropriate washing facility available” (*p* = 0.020) and “It takes too long” (*p* = 0.012) (see appendix, Table A.4). In terms of effect sizes, differences were trivial (d ≤ 0.20) except for “Others might consider it inappropriate” and “I feel that it is not necessary” with small effects (d = 0.28 and d = 0.34, respectively). For the latter, this is comparable to the pre-pandemic difference with d = 0.34.

Multiple logistic regressions reveal that in the 2023 survey the effect of pet ownership on “I feel that it is not necessary”, “I do not have an appropriate washing facility available” and on “Other might consider it inappropriate” is independent of gender, age, educational background, migration background, having children under the age of 16 in the household, having a chronic disease and working in healthcare (*p* < 0.001, *p* = 0.002 and *p* = 0.012, respectively; see Table A.5 in the appendix), like in pre-pandemic data. In 2023, adjusted for all factors, a medium effect of pet ownership was found on “I feel that it is not necessary” (d = 0.31, OR = 1.77, 95%-CI: 1.27; 2.47, *p* < 0.001), “I do not have an appropriate washing facility available” (d = 0.26, OR = 0.63, 95%-CI: 0.48; 0.84, *p* = 0.002), and “Other might consider it inappropriate” (d = 0.36, OR = 0.52, 95%-CI: 0.32; 0.87, *p* = 0.012). For the first two statements, this was similar to pre-pandemic results (d = 0.36, OR = 1.93, 95%-CI: 1.54; 2.41, *p* < 0.001, and d = 0.43, OR = 0.46, 95%-CI: 0.38; 0.56, *p* < 0.001, respectively) [25].

## Discussion

This study replicates analyses of handwashing compliance in different situations among pet owners and non-pet owners at a post-pandemic time point and compares results with pre-pandemic data [25]. Overall, compliance rates increased for the indications “before visiting someone weakened by disease”, “after coming home from outside”, “after being with someone with an infectious disease”, “after blowing one’s nose or coughing into one’s hand”, and “after shaking hands”. In pre-pandemic data, a considerable difference between pet owners and non-pet owners was found in terms of handwashing compliance “after touching animals”. This difference was even larger in the post-pandemic data (2012-19: − 20.2%, d = 0.45; 2023: − 25.9%, d = 0.59). In multiple logistic regression, this difference was found to be independent of possible confounders. The most common reason cited by pet owners and non-pet owners for non-compliance “after touching animals” continued to be “I feel that it is not necessary” (pet owners 78.7%, non-pet owners 66.5%, d = 0.34). In multiple logistic regressions, this difference was found to be independent of possible confounders as well.

As a replication, limitations are similar to the earlier study: animal species and the type of animal contact were not further specified, compliance was defined only by frequency, and reasons for not washing hands were only asked of participants who stated that they rarely or (almost) never washed their hands [25].

Moreover, it should be kept in mind that *self-reported* compliance rates were evaluated, meaning that the true rates may be higher or lower for both pet owners and non-pet owners. During the pandemic, the population in Germany was called upon to comply with several infection prevention measures. Some of these measures, e.g., mask wearing, were enforced and controlled by the authorities. Thus, as an effect of social desirability or reactive defense mechanisms, self-reported rates may have been (deliberately) overestimated by some participants of the post-pandemic survey. At odds with this possibility, a recent study found an increase in self-reported infection prevention and control activities among nurses after the pandemic [37]. Presumably, this increase was influenced by practices routinized in the pandemic. Yet, infection prevention behavior of healthcare professionals and the general population may have been affected differently by the pandemic.

Furthermore, no longitudinal study was evaluated, instead, several cross-sectional samples from different points in time were compared. Thus, no conclusions can be drawn about real changes in terms of pet ownership and handwashing compliance. For instance, the variable “pet ownership” only provides information about whether at least one animal currently lives in the household, but not about whether pet ownership has changed over the years. These aspects could have biased our results. Each of the survey samples was representative of the German adult population, and the weighting was based on the official population update and the micro census of the Federal Statistical Office [29–33]. Table A.1 in the appendix showed some significant differences between the pre-pandemic samples from 2012 to 2019 and the post-pandemic sample from 2023, but these are consistent with general population trends in Germany [38]. Pet ownership did not change significantly, which is in contrast to developments in the United States [39]. Annual representative market research surveys conducted by the German Pet Trade and Industry Association (Zentralverband Zoologischer Fachbetriebe Deutschlands e. V.) and the Industrial Association of Pet Care Producers (Industrieverband Heimtierbedarf e. V.) indicate a higher proportion of pet owners and showed a slight increase in pet ownership during the pandemic, but this proportion fell back to pre-pandemic levels in 2023 (2019: 45%, 2020: 47%, 2021: 47%, 2022: 46%, 2023: 45%) [40–44]. This supports our finding that pet ownership proportion in Germany did not change sustainably in context of the pandemic. Finally, the results cannot necessarily be generalized to other population groups or countries. Pet ownership, especially the type of pet, how it is kept, and what infection prevention measures are available, may vary culturally, and general handwashing compliance rates also differ between countries [45].

Regarding strengths, besides population representativeness and sufficient sample size, this study is (to the best of our knowledge) the first to report pre- and post-pandemic comparisons of hygiene behavior among pet owners and non-pet owners. Comparisons showed an increase in compliance among pet owners *and* non-pet owners, particularly for indications that had been the focus of infection prevention communication during the pandemic.

However, when comparing the rates of all five surveys separately (for 2012-19 see Table A.1 in [25]), differences between pet and non-pet owners emerge. Handwashing compliance for „After being with someone with an infectious disease” varied similarly in 2012-19 among pet owners (2012: 69.7%, 2014: 70.3%, 2017: 75.1%, 2019: 75.6%) *and* among non-pet owners (2012: 69.5%, 2014: 70.8%, 2017: 75.7%, 2019: 75.9%). However, in 2023 non-pet owners peaked at 82.5%, while pet owners stood at 75.5% only. Put differently, in each of the 2012-19 surveys, pet owners and non-pet owners differed only minimally in terms of compliance (0.2%; − 0.6%), while in 2023 the difference was −7.0%. Similar patterns were found for „after coming home from outside” and “after handshaking”. Yet, a different trend emerged regarding “before visiting someone weakened by disease”, as pet owners’ and non-pet owners’ compliance rate *increased nearly equally* in 2023. While in 2012-19 the rates again varied similarly among pet owners and non-pet owners (48.7%; 59.1% and 47.6%; 57.9%, respectively), they *both* increased in 2023 to 68.7% and 69.9%, respectively.

Comparing these two patterns, it is noticeable that in the former situations, handwashing may presumably seem to be more for self-protection than for the protection of others. In contrast, handwashing “before visiting someone weakened by disease” is more about protecting others. This seemed to be equally important to pet owners and non-pet owners in pre-pandemic times, but also *equally more important* to both groups after the pandemic. Handwashing as an act of self-protection, however, seems to be more important for non-pet owners than pet owners, as the higher compliance rates – especially after the pandemic – indicate. While no causal effects of the pandemic can be delineated here, this interpretation at least seems probable. In general, the effectiveness of public health messages for infection prevention behavior has been shown to be predicted by demographic characteristics such as age, gender, health status and ethnicity [46]. In the current analyses the effect of pet ownership on handwashing compliance was found to be independent from such potential confounders. Thus, pet ownership may in fact be another characteristic influencing one’s perception of prosocial and self-focused motivational appeals.

In the pre-pandemic data, the difference between pet owners and non-pet owners in terms of handwashing compliance “after touching animals” was by far the largest across indications [25]. Unexpectedly, it was even larger in 2023 than in the pre-pandemic data (− 25.9% vs. − 20.2%). Again, it is instructive to explore the development of this compliance indicator survey by survey. Both pet and non-pet owners showed a downward trend in compliance rates from 2012 to 2019, however, both declining at different rates. Whereas non-pet owners’ compliance fell by − 2.9% (from 57.3% in 2012 to 54.4% in 2019), pet owners declined by − 7.1% (from 38.6% in 2012 to 31.5% in 2019) [25]. After the pandemic, non-pet owners’ compliance slightly rose again to 55.9%, while it continued to fall to 30.0% among pet owners. That is to say, the pandemic appears to have halted the downward trend among non-pet owners, but not for pet owners.

This is at odds with our assumption that the difference between both groups could have been diminished in the context of the pandemic. However, it aligns with the slight downward trend in pet owners’ compliance found in the UK during the pandemic (see Introduction [24]). This downward trend in handwashing compliance could be explained by pet owners perceiving a reduced risk of infection from their pets since the pandemic. This false belief could have two origins. Firstly, pandemic-related social distancing may have contributed to a stronger bond between pet owners and their animals, which increased anthropomorphism. In fact, this may also increase the risk of infection, as pet owners integrate their animals even more into their personal living space and thus neglect the necessary infection prevention measures [47]. Secondly, pets increased their owners’ mental health and well-being during the pandemic [27, 48, 49], which may have led to a more careless attitude towards infection prevention measures as well. However, both aspects align with the theory of risk perception influencing one’s behavior as stated in various psychological models (e.g., the Health Action Process Approach [50]). Accordingly, a study on predicting health behaviors during the COVID-19 pandemic showed that individual risk perception predicted handwashing [51].

Washing hands “after touching animals” once more may seem to be more about self-protection, and again, this appears to be less important to pet owners, even after the pandemic. This is consistent with the aforementioned fact that pet owners seemed hardly concerned that they could catch COVID-19 from their pet, but rather that their pet could become infected or who would care for the pet if they became ill or even hospitalized [27, 28]. Following this assumption, more communication may be needed about how handwashing after contact with animals, including pets, is not only about self-protection, but serves the protection of others as well: pet owners are more likely to pass germs from their pets to others. Particularly because they have more opportunities for contact with animals, and therefore have a potentially higher risk of transmission. Among infections that are realistically preventable by handwashing are those due to *Salmonella spp.*,* Campylobacter spp.*,* Toxocara canis*, and *Microsporum canis* [52]. However, for pet owners with a higher risk of infection, such as elderly or immunocompromised people, communicating the benefits of handwashing for self-protection seems essential. This holds especially because of evidence that these groups in particular experienced improved mental health and well-being during the pandemic due to pet ownership [53, 54].

Finally, the reason most frequently cited by pet owners and non-pet owners who rarely or (almost) never washed their hands after animal contact remained “I feel that it is not necessary“. Furthermore, it continued to be confirmed more often by pet owners than by non-pet owners even after the pandemic. In 2017 and 2019, all four other reasons were more likely to be cited by non-pet owners. In 2023, this was the opposite for “I don’t think about it or forget it” and “It takes too long”: these two reasons were cited more frequently by pet owners than by non-pet owners. This could indicate that pet owners were somewhat aware that handwashing is indicated after touching animals (even if they did not feel that it is generally necessary), but did not practice it for other reasons. Interestingly, the proportion of pet owners being noncompliant was higher after the pandemic (38.8% in 2017-19 vs. 45.1% in 2023), which was not the case among non-pet owners (19.1% in 2017-19 vs. 17.8% in 2023). This aligns with the aforementioned trend of pet owners becoming less compliant than non-pet owners in the context of the pandemic.

## Conclusion

The initial assumption that the difference between pet owners and non-pet owners in terms of handwashing compliance after touching animals would diminish in the context of the COVID-19 pandemic has been disproved. The gap in self-reported compliance has actually become even greater, mainly due to the lower compliance rate among the pet owners. Non-pet owners showed increased handwashing compliance for the indications that were promoted as a part of national infection prevention communication during the pandemic. In contrast, pet owners showed an increased compliance rate specifically for indications that serve to protect others from infection, but not in situations involving self-protection. For public health efforts during and after pandemics such as COVID-19, this may suggest to make a case for self-protection of pet owners. In this context, the perception of the risks of infection and the asset of pet ownership in terms of not merely companionship, but a source of important resources for one’s health [27, 48, 49] should be explicitly combined.

## Supplementary Information

Supplementary Material 1.

## Data Availability

The 2023 data set was provided by the BIÖG upon our request (Andrea Rückle, personal communication, May 6, 2025).
