# Supplementary material for: Self-reported handwashing frequency among pet and non-pet owners in the German adult population in 2023: a post-pandemic replication study
Source: BMC Public Health. 2026 Apr 7;26:1221. doi: 10.1186/s12889-026-27235-1 (PMC13081525; doi:10.1186/s12889-026-27235-1)
Supplement: Supplementary file 1 — Supplementary Material 1. [file 12889_2026_27235_MOESM1_ESM.docx]

**Table A.1** Sample characteristics, overall and stratified for pre- and post-pandemic survey samples

|  | |  | **Total**  **N=19,156 (100%)** | | |  | **Pre-pandemic  samples (2012-2019) (N=15,559, 81.2%)** | | |  | **Post-pandemic sample (2023) (N=3,597, 18.8%)** | | |  | **Chi²-test****** | | |
| --- | --- | --- | --- | --- | --- | --- | --- | --- | --- | --- | --- | --- | --- | --- | --- | --- | --- |
|  | |  | N* |  | %** |  | N |  | %*** |  | N |  | %*** |  | Chi² |  | *p* |
| *Gender* | | | | | | | | | |  |  |  |  |  | 0.05 |  | 0.817 |
|  | Men |  | 9,428 |  | 49.2% |  | 7,651 |  | 49.2% |  | 1,777 |  | 49.4% |  |  |  |  |
|  | Women |  | 9,728 |  | 50.8% |  | 7,908 |  | 50.8% |  | 1,820 |  | 50.6% |  |  |  |  |
| *Age (in years)* | | | | | | | | | |  |  |  |  |  | 9.7 |  | 0.021 |
|  | 16 – 29 |  | 3,837 |  | 20.0% |  | 3,140 |  | 20.2% |  | 697 |  | 19.4% |  |  |  |  |
|  | 30 – 44 |  | 4,521 |  | 23.6% |  | 3,652 |  | 23.5% |  | 869 |  | 24.2% |  |  |  |  |
|  | 45 – 59 |  | 5,359 |  | 28.0% |  | 4,409 |  | 28.3% |  | 950 |  | 26.4% |  |  |  |  |
|  | 60 – 85 |  | 5,439 |  | 28.4% |  | 4,358 |  | 28.0% |  | 1,081 |  | 30.1% |  |  |  |  |
| *Educational background* ^a^ | | | | | | | | | |  |  |  |  |  | 75.0 |  | <0.001 |
|  | Higher |  | 6,369 |  | 33.7% |  | 5,016 |  | 32.5% |  | 1,353 |  | 39.0% |  |  |  |  |
|  | Medium |  | 5,905 |  | 31.2% |  | 4,808 |  | 31.1% |  | 1,097 |  | 31.6% |  |  |  |  |
|  | Lower |  | 6,647 |  | 35.1% |  | 5,624 |  | 36.4% |  | 1,023 |  | 29.5% |  |  |  |  |
| *Migration background* | | | | | | | | | |  |  |  |  |  | 4.8 |  | 0.029 |
|  | No |  | 15,246 |  | 79.7% |  | 12,430 |  | 79.9% |  | 2,816 |  | 78.3% |  |  |  |  |
|  | Yes |  | 3,907 |  | 20.3% |  | 3,126 |  | 20.1% |  | 781 |  | 21.7% |  |  |  |  |
| *Children under 16 in household* | | | | | | | | | |  |  |  |  |  | 0.02 |  | 0.881 |
|  | No |  | 14,541 |  | 76.5% |  | 11,808 |  | 76.5% |  | 2,733 |  | 76.4% |  |  |  |  |
|  | Yes |  | 4,473 |  | 23.5% |  | 3,628 |  | 23.5% |  | 845 |  | 23.6% |  |  |  |  |
| *Children age 5 or younger in household* | | | | | | | | | |  |  |  |  |  | 0.6 |  | 0.453 |
|  | No |  | 17,145 |  | 90.7% |  | 13,924 |  | 90.7% |  | 3,221 |  | 90.3% |  |  |  |  |
|  | Yes |  | 1,768 |  | 9.3% |  | 1,422 |  | 9.3% |  | 346 |  | 9.7% |  |  |  |  |
| *Chronic disease* | | | | | | | | | |  |  |  |  |  | 9.8 |  | 0.002 |
|  | No |  | 13,488 |  | 70.6% |  | 11,029 |  | 71.1% |  | 2,459 |  | 68.4% |  |  |  |  |
|  | Yes |  | 5,624 |  | 29.4% |  | 4,490 |  | 28.9% |  | 1,134 |  | 31.6% |  |  |  |  |
| *Currently working in healthcare ^b^* | | | | | | | | | |  |  |  |  |  | 92.0 |  | <0.001 |
|  | No |  | 17,350 |  | 90.6% |  | 14,068 |  | 90.5% |  | 3,282 |  | 91.3% |  |  |  |  |
|  | Yes |  | 1,796 |  | 9.4% |  | 1,485 |  | 9.5% |  | 311 |  | 8.7% |  |  |  |  |
| *Pet ownership* | | | | | | | | | |  |  |  |  |  | 0.9 |  | 0.335 |
|  | No |  | 12,060 |  | 63.0% |  | 9,821 |  | 63.1% |  | 2,239 |  | 62.2% |  |  |  |  |
|  | Yes |  | 7,096 |  | 37.0% |  | 5,738 |  | 36.9% |  | 1,358 |  | 37.8% |  |  |  |  |

*Notes.* * Any data not adding to the total is due to missing values ** Column percentages *** Row percentages **** Pet ownership by row variable
^a^ Higher educational background equals upper secondary school, middle educational background equals intermediate school, and lower educational background equals secondary general school
^b^ Pertains to the survey participants personally

Data from 2012-19 originally published in [25]

**Table A.2** Self-reported handwashing compliance as proportions (in %) of those reporting to always or almost always wash their hands, for nine different indications
 (overall and for pet owners and non-pet owners)

|  | |  | **Total** | | |  | **Non-pet owners** | | |  | **Pet owners** | | |  | | **∆^a^** |  | **Chi²-test** | | | | | | |  | **Breslow-Day-Test^b^** |
| --- | --- | --- | --- | --- | --- | --- | --- | --- | --- | --- | --- | --- | --- | --- | --- | --- | --- | --- | --- | --- | --- | --- | --- | --- | --- | --- |
|  | |  | N |  | % |  | N |  | % |  | N |  | % |  |  | |  | Chi² |  | *p* |  | OR |  | 95% CI |  |  |
| **After using the toilet** | | |  | | | | | | | | | | | | |  |  |  |  |  |  |  |  |  |  |  |
|  | 2012-2019 |  | 14,886 |  | 95.7% |  | 9,388 |  | 95.6% |  | 5,498 |  | 95.8% |  | 0.2% | |  | 0.35 |  | 0.554 |  | 1.05 |  | 0.89; 1.23 |  | Chi²=0.0002  *p*=0.988 |
|  | 2023 |  | 3,479 |  | 96.7% |  | 2,164 |  | 96.7% |  | 1,315 |  | 96.8% |  | 0.1% | |  | 0.09 |  | 0.765 |  | 1.06 |  | 0.72; 1.55 |  |  |
| **Before handling food** | | |  | | | | | | | | | | | | |  |  |  |  |  |  |  |  |  |  |  |
|  | 2012-2019 |  | 12,734 |  | 81.8% |  | 7,910 |  | 80.5% |  | 4,824 |  | 84.1% |  | 3.6% | |  | 30.50 |  | <0.001 |  | 1.28 |  | 1.17; 1.39 |  | Chi²=0.32  *p*=0.569 |
|  | 2023 |  | 2,907 |  | 80.8% |  | 1,786 |  | 79.8% |  | 1,121 |  | 82.6% |  | 2.8% | |  | 4.40 |  | 0.036 |  | 1.21 |  | 1.01; 1.43 |  |  |
| **After being with someone with**  **an infectious disease** | | |  | | | | | | | | | | | | |  |  |  |  |  |  |  |  |  |  |  |
|  | 2012-2019 |  | 11,311 |  | 72.7% |  | 7,153 |  | 72.8% |  | 4,158 |  | 72.5% |  | -0.3% | |  | 0.22 |  | 0.637 |  | 0.98 |  | 0.91; 1.06 |  | Chi²=20.00  *p*<0.001 |
|  | 2023 |  | 2,872 |  | 79.9% |  | 1,848 |  | 82.5% |  | 1,024 |  | 75.5% |  | -7.0% | |  | 26.31 |  | <0.001 |  | 0.65 |  | 0.55; 0.77 |  |  |
| **Before visiting someone**  **weakened by illness** | | |  | | | | | | | | | | | | |  |  |  |  |  |  |  |  |  |  |  |
|  | 2012-2019 |  | 8,324 |  | 53.5% |  | 5,193 |  | 52.9% |  | 3,131 |  | 54.6% |  | 1.7% | |  | 4.20 |  | 0.041 |  | 1.07 |  | 1.01; 1.14 |  | Chi²=2.42  *p*=0.120 |
|  | 2023 |  | 2,498 |  | 69.5% |  | 1,566 |  | 69.9% |  | 932 |  | 68.7% |  | -1.2% | |  | 0.63 |  | 0.426 |  | 0.94 |  | 0.81; 1.09 |  |  |
| **After coming home from outside** | | |  | | | | | | | | | | | | |  |  |  |  |  |  |  |  |  |  |  |
|  | 2012-2019 |  | 7,412 |  | 47,6% |  | 4,801 |  | 48.9% |  | 2,611 |  | 45.5% |  | -3.4% | |  | 16.70 |  | <0.001 |  | 0.87 |  | 0.82; 0.3 |  | Chi²=12.19  *p*<0.001 |
|  | 2023 |  | 2,041 |  | 56.8% |  | 1,355 |  | 60.5% |  | 686 |  | 50.6% |  | -9.9% | |  | 34.19 |  | <0.001 |  | 0.67 |  | 0.58; 0.76 |  |  |
| **Before eating** | | |  | | | | | | | | | | | | |  |  |  |  |  |  |  |  |  |  |  |
|  | 2012-2019 |  | 9,364 |  | 60.2% |  | 5,824 |  | 59.3% |  | 3,540 |  | 61.7% |  | 2.4% | |  | 8.70 |  | 0.003 |  | 1.11 |  | 1.03; 1.18 |  | Chi²=0.65  *p*=0.422 |
|  | 2023 |  | 2,026 |  | 56.3% |  | 1,253 |  | 56.0% |  | 773 |  | 56.9% |  | 0.9% | |  | 0.32 |  | 0.574 |  | 1.04 |  | 0.91; 1.19 |  |  |
| **After touching animals** | | |  | | | | | | | | | | | | |  |  |  |  |  |  |  |  |  |  |  |
|  | 2012-2019 |  | 7,506 |  | 48.3% |  | 5,467 |  | 55.7% |  | 2,039 |  | 35.5% |  | -20.2% | |  | 588.30 |  | <0.001 |  | 0.44 |  | 0.41; 0.47 |  | Chi²=10.65  *p*=0.001 |
|  | 2023 |  | 1,659 |  | 46.1% |  | 1,252 |  | 55.9% |  | 407 |  | 30.0% |  | -25.9% | |  | 229.02 |  | <0.001 |  | 0.34 |  | 0.29; 0.39 |  |  |
| **After blowing nose or coughing  in one's hand** | | |  | | | | | | | | | | | |  | |  |  |  |  |  |  |  |  |  |  |
|  | 2012-2019 |  | 4,563 |  | 29.3% |  | 2,816 |  | 28.7% |  | 1,747 |  | 30.5% |  | 1.8% | |  | 5.50 |  | 0.019 |  | 1.09 |  | 1.01; 1.17 |  | Chi²=2.10  *p*=0.147 |
|  | 2023 |  | 1,229 |  | 34.2% |  | 771 |  | 34.5% |  | 458 |  | 33.7% |  | -0.8% | |  | 0.20 |  | 0.657 |  | 0.97 |  | 0.84; 1.12 |  |  |
| **After handshaking** | | |  |  |  |  |  |  |  |  |  |  |  |  |  | |  |  |  |  |  |  |  |  |  |  |
|  | 2012-2019 |  | 1,077 |  | 6.9% |  | 728 |  | 7.4% |  | 349 |  | 6.1% |  | -1.3% | |  | 9.90 |  | 0.002 |  | 0.81 |  | 0.71; 0.92 |  | Chi²=0.33  *p*=0.568 |
|  | 2023 |  | 419 |  | 11.7% |  | 285 |  | 12.7% |  | 134 |  | 9.9% |  | -2.8% | |  | 6.69 |  | 0.010 |  | 0.75 |  | 0.60; 0.93 |  |  |

Notes: ^a^ Percentage difference between pet and non-pet owners, ^b^ across surveys

Data from 2012-19 originally published in [25]

**Table A.3** Results of multiple logistic regression analyses for handwashing compliance in different situations (indications)^§^

|  | **After using the toilet** | | | **Before handling food** | | | **After being with**  **someone with an**  **infectious disease** | | | **Before visiting someone weakened by illness** | | | **After coming home from outside** | | | **Before eating** | | | **After touching animals** | | | **After blowing nose or coughing in one’s hand** | | | **After handshaking** | | |
| --- | --- | --- | --- | --- | --- | --- | --- | --- | --- | --- | --- | --- | --- | --- | --- | --- | --- | --- | --- | --- | --- | --- | --- | --- | --- | --- | --- |
|  | **OR** | **95%-CI** | **p** | **OR** | **95%-CI** | **p** | **OR** | **95%-CI** | **p** | **OR** | **95%-CI** | **p** | **OR** | **95%-CI** | **p** | **OR** | **95%-CI** | **p** | **OR** | **95%-CI** | **p** | **OR** | **95%-CI** | **p** | **OR** | **95%-CI** | **p** |
| **Pet ownership** |  | | |  | | |  | | |  | | |  | | |  | | |  |  |  |  |  |  |  |  |  |
| Yes | 0.97 | 0.64-1.45 | =  0.887 | 0.95 | 0.81-1.11 | =  0.514 | 0.63 | 0.55-0.73 | <  0.001 | 1.19 | 0.99-1.43 | =  0.070 | 0.66 | 0.55-0.79 | <  0.001 | 1.08 | 0.93-1.25 | =  0.321 | 0.34 | 0.29-0.40 | <  0.001 | 0.96 | 0.83-1.12 | =  0.636 | 0.86 | 0.68-1.08 | =  0.188 |
| No | ref. |  |  | ref. |  |  | ref. |  |  | ref. |  |  | ref. |  |  | ref. |  |  | ref. |  |  | ref. |  |  | ref. |  |  |
| **Gender** |  | | |  | | |  | | |  | | |  | | |  | | |  |  |  |  |  |  |  |  |  |
| Women | 1.49 | 1.01-2.19 | =  0.047 | 1.94 | 1.67-2.26 | <  0.001 | 2.35 | 2.03-2.71 | <  0.001 | 1.39 | 1.16-1.65 | <  0.001 | 1.58 | 1.33-1.89 | <  0.001 | 0.97 | 0.84-1.11 | =  0.621 | 1.42 | 1.23-1.65 | <  0.001 | 1.59 | 1.37-1.84 | <  0.001 | 1.38 | 1.11-1.71 | =  0.004 |
| Men | ref. |  |  | ref. |  |  | ref. |  |  | ref. |  |  | ref. |  |  | ref. |  |  | ref. |  |  | ref. |  |  |  |  |  |
| **Age** |  | | |  | | |  | | |  | | |  | | |  | | |  |  |  |  |  |  |  |  |  |
| 60-85 years of age | 0.85 | 0.48-1.52 | =  0.588 | 0.99 | 0.78-1.25 | =  0.916 | 1.04 | 0.83-1.31 | =  0.709 | 0.68 | 0.52-0.90 | =  0.008 | 1.39 | 1.06-1.83 | =  0.018 | 1.60 | 1.28-2.00 | <  0.001 | 2.98 | 2.34-3.79 | <  0.001 | 0.65 | 0.51-0.82 | <  0.001 | 1.38 | 0.96-2.00 | =  0.082 |
| 45-59 years of age | 2.10 | 1.09-4.04 | =  0.027 | 0.99 | 0.78-1.25 | =  0.923 | 1.32 | 1.06-1.65 | =  0.014 | 0.79 | 0.60-1.04 | =  0.096 | 1.29 | 0.99-1.68 | =  0.058 | 1.44 | 1.16-1.78 | =  0.001 | 2.30 | 1.82-2.91 | <  0.001 | 0.83 | 0.66-1.05 | =  0.121 | 0.92 | 0.63-1.34 | =  0.644 |
| 30-44 years of age | 1.38 | 0.75-2.53 | =  0.296 | 1.04 | 0.81-1.33 | =  0.749 | 1.24 | 0.98-1.57 | =  0.074 | 0.93 | 0.69-1.25 | =  0.612 | 1.15 | 0.88-1.51 | =  0.317 | 1.21 | 0.96-1.53 | =  0.101 | 1.82 | 1.42-2.33 | <  0.001 | 1.17 | 0.92-1.50 | =  0.205 | 1.42 | 0.96-2.11 | =  0.078 |
| 16-29 years of age | ref. |  |  | ref. |  |  | ref. |  |  | ref. |  |  | ref. |  |  | ref. |  |  | ref. |  |  | ref. |  |  | ref. |  |  |
| **Educational background**** |  | | |  | | |  | | |  | | |  | | |  | | |  |  |  |  |  |  |  |  |  |
| lower | 0.99 | 0.61-1.59 | =  0.960 | 1.39 | 1.14-1.70 | =  0.001 | 1.43 | 1.19-1.73 | <  0.001 | 1.67 | 1.33-2.11 | <  0.001 | 1.17 | 0.93-1.46 | =  0.179 | 1.80 | 1.50-2.16 | <  0.001 | 1.27 | 1.06-1.54 | =  0.012 | 2.37 | 1.96-2.88 | <  0.001 | 2.30 | 1.73-3.05 | <  0.001 |
| intermediate | 1.92 | 1.15-3.20 | =  0.013 | 0.99 | 0.83-1.18 | =  0.904 | 1.21 | 1.02-1.44 | =  0.028 | 1.45 | 1.18-1.80 | <  0.001 | 1.38 | 1.12-1.71 | =  0.003 | 1.37 | 1.16-1.61 | <  0.001 | 1.12 | 0.94-1.34 | =  0.207 | 1.51 | 1.25-1.81 | <  0.001 | 1.44 | 1.08-1.92 | =  0.014 |
| higher | ref. |  |  | ref. |  |  | ref. |  |  | ref. |  |  | ref. |  |  | ref. |  |  | ref. |  |  | ref. |  |  | ref. |  |  |
| **Migration background** |  | | |  | | |  | | |  | | |  | | |  | | |  |  |  |  |  |  |  |  |  |
| Yes | 1.31 | 0.79-2.17 | =  0.290 | 0.91 | 0.76-1.09 | =  0.307 | 1.93 | 1.61-2.31 | <  0.001 | 1.49 | 1.18-1.88 | <  0.001 | 1.08 | 0.80-1.34 | =  0.461 | 1.59 | 1.34-1.90 | <  0.001 | 1.34 | 1.13-1.60 | =  0.001 | 1.26 | 1.05-1.50 | =  0.011 | 1.55 | 1.22-1.98 | <  0.001 |
| No | ref. |  |  | ref. |  |  | ref. |  |  | ref. |  |  | ref. |  |  | ref. |  |  | ref. |  |  | ref. |  |  | ref. |  |  |
| **Children under 16 years of age in household** |  | | |  | | |  | | |  | | |  | | |  | | |  |  |  |  |  |  |  |  |  |
| Yes | 0.50 | 0.30-0.84 | =  0.009 | 1.06 | 0.86-1.31 | =  0.565 | 1.01 | 0.83-1.23 | =  0.909 | 1.05 | 0.82-1.35 | =  0.684 | 0.91 | 0.73-1.15 | =  0.447 | 1.09 | 0.90-1.32 | =  0.386 | 1.37 | 1.12-1.67 | =  0.002 | 0.81 | 0.66-1.00 | =  0.049 | 0.56 | 0.40-0.80 | =  0.001 |
| No | ref. |  |  | ref. |  |  | ref. |  |  | ref. |  |  | ref. |  |  | ref. |  |  | ref. |  |  | ref. |  |  | ref. |  |  |
| **Chronic disease** |  | | |  | | |  | | |  | | |  | | |  | | |  |  |  |  |  |  |  |  |  |
| Yes | 1.46 | 0.93-2.29 | =  0.104 | 0.98 | 0.83-1.16 | =  0.811 | 1.06 | 0.91-1.24 | =  0.474 | 1.03 | 0.85-1.24 | =  0.779 | 1.12 | 0.92-1.36 | =  0.249 | 0.91 | 0.78-1.06 | =  0.210 | 1.26 | 1.08-1.47 | =  0.004 | 1.27 | 1.09-1.49 | =  0.003 | 1.31 | 1.05-1.63 | =  0.019 |
| No | ref. |  |  | ref. |  |  | ref. |  |  | ref. |  |  | ref. |  |  | ref. |  |  | ref. |  |  | ref. |  |  | ref. |  |  |
| **Currently working in healthcare** |  | | |  | | |  | | |  | | |  | | |  | | |  |  |  |  |  |  |  |  |  |
| Yes | 1.14 | 0.53-2.42 | =  0.744 | 1.38 | 1.03-1.83 | =  0.029 | 1.28 | 0.99-1.67 | =  0.061 | 1.03 | 0.75-1.42 | =  0.851 | 2.31 | 1.57-3.41 | <  0.001 | 1.23 | 0.96-1.57 | =  0.108 | 1.18 | 0.91-1.52 | =  0.214 | 1.66 | 1.30-2.13 | <  0.001 | 1.12 | 0.75-1.66 | =  0.586 |
| No | ref. |  |  | ref. |  |  | ref. |  |  | ref. |  |  | ref. |  |  | ref. |  |  | ref. |  |  | ref. |  |  | ref. |  |  |

Notes: §OR: odds ratio; CI: confidence interval; all estimates are from the multiple logistic regression model for each respective behavioral indicator, which included all predictors listed **lower: secondary general school, intermediate: middle school, higher: upper secondary school

**Table A.4** Participants’ reasons for (almost) never or rarely washing hands after touching animals*, **

|  |  | **Total** | | |  | **Non-pet owners** | | |  | **Pet owners** | | |  | **∆^a^** |  | **Chi²-test** | | | | | | | | | **Breslow-Day-Test^b^** |
| --- | --- | --- | --- | --- | --- | --- | --- | --- | --- | --- | --- | --- | --- | --- | --- | --- | --- | --- | --- | --- | --- | --- | --- | --- | --- |
|  |  | N |  | % |  | N |  | % |  | N |  | % |  |  |  | Chi² |  | *p* |  | OR |  | 95% CI | |  |  |
| **I feel that it is not necessary.** | |  |  |  |  |  |  |  |  |  |  |  |  |  |  |  |  |  |  |  |  |  | |  |  |
| *2017-2019* | | 1,413 |  | 73.5% |  | 597 |  | 67.1% |  | 816 |  | 79.0% |  | 11.9% |  | 34.83 |  | <0.001 |  | 1.85 |  | 1.50; 2.27 | |  | Chi²=0.001  *p*=0.970 |
| *2023* | | 730 |  | 73.9% |  | 258 |  | 66.5% |  | 472 |  | 78.7% |  | 12.2% |  | 18.09 |  | <0.001 |  | 1.86 |  | 1.39; 2.48 | |  |  |
| **I do not think of it, or forget it.** | |  |  |  |  |  |  |  |  |  |  |  |  |  |  |  |  |  |  |  |  |  | |  |  |
| *2017-2019* | | 1,130 |  | 58.7% |  | 558 |  | 62.6% |  | 572 |  | 55.3% |  | -7.3% |  | 10.70 |  | 0.001 |  | 0.74 |  | 0.61; 0.89 | |  | Chi²=6.35  *p*=0.012 |
| *2023* | | 562 |  | 56.3% |  | 213 |  | 54.8% |  | 349 |  | 57.2% |  | 2.4% |  | 0.58 |  | 0.445 |  | 1.11 |  | 0.86; 1.43 | |  |  |
| **I do not have an appropriate washing facility available.** | |  |  |  |  |  |  |  |  |  |  |  |  |  |  |  |  |  |  |  |  |  | |  |  |
| *2017-2019* | | 1,021 |  | 53.0% |  | 561 |  | 63.0% |  | 460 |  | 44.5% |  | -18.5% |  | 65.59 |  | <0.001 |  | 0.47 |  | 0.39; 0.57 | |  | Chi²=5.45  *p*=0.020 |
| *2023* | | 516 |  | 51.8% |  | 223 |  | 57.5% |  | 293 |  | 48.1% |  | -9.4% |  | 8.32 |  | 0.004 |  | 0.69 |  | 0.53; 0.89 | |  |  |
| **It takes too long.** | |  |  |  |  |  |  |  |  |  |  |  |  |  |  |  |  |  |  |  |  |  |  | |  |
| *2017-2019* | | 288 |  | 15.0% |  | 151 |  | 16.9% |  | 137 |  | 13.3% |  | -3.6% |  | 5.10 |  | 0.024 |  | 0.75 |  | 0.58; 0.96 | |  | Chi²=6.30  *p*=0.012 |
| *2023* | | 149 |  | 14.9% |  | 50 |  | 12.8% |  | 99 |  | 16.3% |  | 3.5% |  | 2.26 |  | 0.133 |  | 1.32 |  | 0.92; 1.91 | |  |  |
| **Others might consider it inappropriate.** | |  |  |  |  |  |  |  |  |  |  |  |  |  |  |  |  |  |  |  |  |  |  | |  |
| *2017-2019* | | 262 |  | 13.6% |  | 136 |  | 15.2% |  | 126 |  | 12.2% |  | -3.0% |  | 3.82 |  | 0.051 |  | 0.77 |  | 0.59; 1.00 | |  | Chi²=0.79  *p*=0.373 |
| *2023* | | 86 |  | 8.6% |  | 43 |  | 11.1% |  | 43 |  | 7.1% |  | -4.0% |  | 4.83 |  | 0.028 |  | 0.61 |  | 0.39; 0.95 | |  |  |

Notes: *Multiple responses were possible. ** Reasons only available for participants who stated to never, almost never or rarely wash hands after touching an animal;
 ^a^ Percentage difference between pet owners and non-pet owner, ^b^ across surveys

Data from 2017-19 originally published in [25]

**Table A.5** Results of multiple logistic regression analyses for reasons for (almost) never or rarely washing ones hands after touching animals^§^

|  | **I feel that it is not necessary.** | | | **I do not think of it, or forget it.** | | | **I do not have an appropriate washing facility available.** | | | **It takes too long.** | | | **Others might consider it inappropriate.** | | |  |
| --- | --- | --- | --- | --- | --- | --- | --- | --- | --- | --- | --- | --- | --- | --- | --- | --- |
|  | **OR** | **95%-CI** | **p** | **OR** | **95%-CI** | **p** | **OR** | **95%-CI** | **p** | **OR** | **95%-CI** | **p** | **OR** | **95%-CI** | **p** |  |
| **Pet ownership** |  | | | | | | | | | | | | | | | |
| Yes | 1.77 | 1.27-  2.47 | <  0.001 | 1.26 | 0.95-  1.67 | =  0.114 | 0.63 | 0.48-  0.84 | =  0.002 | 1.42 | 0.94-  2.15 | =  0.096 | 0.52 | 0.32-  0.87 | =  0.012 |  |
| No | ref. |  |  | ref. |  |  | ref. |  |  | ref. |  |  | ref. |  |  |  |
| **Gender** |  | | | | | | | | | | | | | | | |
| Women | 1.02 | 0.74-  1.41 | =  0.903 | 0.74 | 0.56-  0.97 | =  0.031 | 1.28 | 0.98-  1.69 | =  0.075 | 0.62 | 0.42-  0.92 | =  0.019 | 1.08 | 0.66-  1.75 | =  0.772 |  |
| Men | ref. |  |  | ref. |  |  | ref. |  |  | ref. |  |  | ref. |  |  |  |
| **Age** |  | | | | | | | | | | | | | | | |
| 60-85 years of age | 0.82 | 0.52-  1.30 | =  0.390 | 0.66 | 0.44-  1.00 | =  0.050 | 0.59 | 0.39-0.88 | =  0.011 | 0.59 | 0.32-  1.09 | =  0.090 | 1.39 | 0.70-  2.78 | =  0.350 |  |
| 45-59 years of age | 0.96 | 0.63-  1.47 | =  0.856 | 0.45 | 0.31-  0.66 | <  0.001 | 0.95 | 0.66-  1.37 | =  0.788 | 0.60 | 0.35-  1.01 | =  0.055 | 1.11 | 0.58-  2.12 | =  0.757 |  |
| 30-44 years of age | 2.25 | 1.32-  3.84 | =  0.003 | 0.68 | 0.45-  1.03 | =  0.068 | 1.89 | 1.23-  2.82 | =  0.003 | 1.03 | 0.61-  1.73 | =  0.923 | 0.37 | 0.16-  0.88 | =  0.024 |  |
| 16-29 years of age | ref. |  |  | ref. |  |  | ref. |  |  | ref. |  |  | ref. |  |  |  |
| **Educational background**** |  | | | | | | | | | | | | | | | |
| lower | 0.46 | 0.31-  0.69 | <  0.001 | 0.56 | 0.39-  0.79 | =  0.001 | 1.40 | 0.98-  2.00 | =  0.068 | 0.52 | 0.29-  0.91 | =  0.023 | 1.30 | 0.69-  2.44 | =  0.418 |  |
| intermediate | 1.33 | 0.90-  1.96 | =  0.159 | 0.88 | 0.65-  1.21 | =  0.431 | 1.03 | 0.76-  1.41 | =  0.842 | 1.15 | 0.76-  1.75 | =  0.504 | 1.28 | 0.72-  2.27 | =  0.401 |  |
| higher | ref. |  |  | ref. |  |  | ref. |  |  | ref. |  |  | ref. |  |  |  |
| **Migration background** |  | | | | | | | | | | | | | | | |
| Yes | 0.43 | 0.30-  0.62 | <  0.001 | 1.15 | 0.82-  1.62 | =  0.412 | 0.97 | 0.69-  1.36 | =  0.843 | 1.49 | 0.96-  2.34 | =  0.078 | 1.12 | 0.62-  2.00 | =  0.709 |  |
| No | ref. |  |  | ref. |  |  | ref. |  |  | ref. |  |  | ref. |  |  |  |
| **Children under 16 years of age in household** |  | | | | | | | | | | | | | | | |
| Yes | 0.95 | 0.60-  1.50 | =  0.813 | 0.94 | 0.65-  1.35 | =  0.725 | 0.76 | 0.52-  1.09 | =  0.135 | 1.05 | 0.65-  1.71 | =  0.832 | 2.10 | 1.10-  4.03 | =  0.025 |  |
| No | ref. |  |  | ref. |  |  | ref. |  |  | ref. |  |  | ref. |  |  |  |
| **Chronic disease** |  | | | | | | | | | | | | | | | |
| Yes | 1.33 | 0.92-  1.92 | =  0.135 | 1.05 | 0.78-  1.43 | =  0.740 | 0.78 | 0.58-  1.06 | =  0.116 | 1.22 | 0.79-  1.90 | =  0.369 | 0.87 | 0.50-  1.52 | =  0.628 |  |
| No | ref. |  |  | ref. |  |  | ref. |  |  | ref. |  |  | ref. |  |  |  |
| **Currently working in healthcare** |  | | | | | | | | | | | | | | | |
| Yes | 0.72 | 0.41-  1.30 | =  0.276 | 0.65 | 0.41-  1.03 | =  0.065 | 0.59 | 0.37-  0.94 | =  0.025 | 0.98 | 0.51-  1.85 | =  0.937 | 0.52 | 0.17-  1.61 | =  0.258 |  |
| No | ref. |  |  | ref. |  |  | ref. |  |  | ref. |  |  | ref. |  |  |  |

Notes: § OR: odds ratio; CI: confidence interval; all estimates are from the multiple logistic regression model for each respective behavioral indicator, which included all predictors listed **lower: secondary general school, intermediate: middle school, higher: upper secondary school
